# Accuracy in the Estimation of Self-Reported Knee Brace Wear Time in Young Adults With a Symptomatic Knee Following ACL Reconstruction: Secondary Analysis of a Pilot Randomized Controlled Trial

**DOI:** 10.2196/79725

**Published:** 2026-05-27

**Authors:** Matthew Savage, Benjamin F Mentiplay, Harriette Slater, Fernanda Serighelli, David L Carey, Jamon L Couch, Andrea M Bruder, Adam G Culvenor

**Affiliations:** 1 La Trobe Sport and Exercise Medicine Research Centre, School of Allied Health, Human Services and Sport, La Trobe University, Plenty Rd, Bundoora, Melbourne, Victoria, 3086, Australia, +61 3 9479 5116; 2 Australian IOC Research Centre, La Trobe University, Melbourne, Australia; 3 Sport and Exercise Science, School of Allied Health, Human Services and Sport, La Trobe University, Melbourne, Australia; 4 Arthritis Research Canada, Vancouver, BC, Canada; 5 Department of Physiotherapy, Podiatry and Prosthetics and Orthotics, School of Allied Health, Human Services and Sport, La Trobe University, Melbourne, Australia

**Keywords:** rehabilitation, knee brace, knee osteoarthritis, physiotherapy, knee injury, sensor, wearable technology

## Abstract

**Background:**

Knee braces may improve symptoms and physical function following anterior cruciate ligament reconstruction (ACLR). However, their effectiveness depends on adherence, which typically relies on self-reported wear time, prone to recall and response bias. Objective measures (eg, temperature sensors), validated in footwear and orthotics research, offer a potentially more accurate alternative to self-reporting. Despite this, there is no research comparing self-reported and sensor-measured wear times in a knee brace.

**Objective:**

This study aimed to determine how well self-reported wear times reflect sensor-measured data in a slim-fit knee brace.

**Methods:**

Young adults (aged 18-45 years), 1-8 years post-ACLR, with a symptomatic knee (the 4 Knee injury and Osteoarthritis Outcome Score subscales [KOOS_4_] score <80/100) wore a slim-fit brace during a 6-week feasibility trial. This study reports a secondary analysis of participants allocated to the brace group. Self-reported wear times were recorded in daily logs. An undisclosed, embedded temperature sensor recorded temperature every 10 minutes. A wear detection algorithm identified brace donning and doffing. These data were used to calculate aggregated measures (ie, summary measures across the entire 6-week intervention period, including cumulative wear time, average daily wear time, and total number of days worn) and repeated measures (daily wear duration, 3- and 7-day rolling averages, where wear time is averaged over consecutive days). Agreement between self-reported and sensor-based measures was assessed using concordance correlation coefficients (CCCs) and limits of agreement (LoA).

**Results:**

Of the 14 randomized participants, 10 (30% male [n=3]; mean age: 33, SD 6 years; time post-ACLR: 4, SD 1 years) had both temperature sensor and self-reported wear data. Six participants (60%) underreported average daily wear time (mean 29, SD 24 minutes across all 10 participants), while nine (90%) overreported the number of days worn (mean 9, SD 6 days across all 10 participants). Daily wear time showed moderate agreement between the sensor and self-reporting (CCC 0.70, 95% CI 0.58-0.79), but wide LoA (−223 to 217 minutes). Using 3- or 7-day rolling averages narrowed LoA (−47 to 36 minutes per day and −14 to 10 minutes per day, respectively) and slightly improved CCCs (0.74, 95% CI 0.58-0.85, and 0.73, 95% CI 0.51-0.86, respectively). Greater agreement was observed with more aggregated outcomes; for total 6-week wear time, the CCC was 0.84 (95%CI 0.50-0.95). When expressed as daily average wear time, the CCC was excellent (0.92, 95% CI 0.73-0.98), although daily LoA remained wide (−68 to 32 minutes), indicating substantial individual variability between self-reported and sensor-based measures. For the total number of days worn, the CCC was moderate (0.64, 95% CI 0.15-0.88) and LoA was wide (−10 to 22 days).

**Conclusions:**

Self-reported daily brace wear time is inaccurate compared to wear time measured by the temperature sensor. Aggregated data and rolling averages showed better agreement. Future intervention studies should consider objective adherence measures. Failing this, averaging self-reported wear time across the intervention period could improve accuracy.

## Introduction

Slim-fit knee braces can improve pain and function after anterior cruciate ligament reconstruction (ACLR) [1,2], but adherence to wearing them is critical for these benefits [3]. Most studies use self-reported measures of adherence [1-5], which are prone to recall, response, and social desirability bias [6] and missing data [3]. Objective measures are needed to track wear time accurately [4]. One promising, low-cost method to objectively measure adherence (ie, wear time) is with an embedded temperature sensor. Such sensors are highly accurate at detecting wear times in footwear and orthotics when compared to reference standard data (ie, camera [7], smartphone application [8]). However, no research compares self-reported and sensor-measured wear times in a knee brace, which has implications for evaluating adherence in intervention studies. Without accurate adherence data, the effectiveness of brace interventions may be misestimated. We aimed to determine how well self-reported wear times reflect temperature sensor-measured data in a slim-fit knee brace.

## Methods

### Ethical Considerations

Ethics approval was granted by the La Trobe University Ethics Committee (HEC23249) and written informed consent was gained from all participants prior to enrollment in the study. This trial involved limited disclosure, where participants in the brace group were not informed of the presence of a small temperature sensor embedded in the brace. As part of our ethical approval, all participants were fully debriefed about the sensor at the end of the trial, where a debriefing form was signed. All participant data were deidentified prior to analysis. Identifiable information was stored separately from study data on secure, password-protected servers accessible only to the research team. Participants received an AUD $25 (USD $18.05) gift voucher for their time, and the knee brace used in the study was provided to participants at no cost.

### Study Design

**Figure 1.**
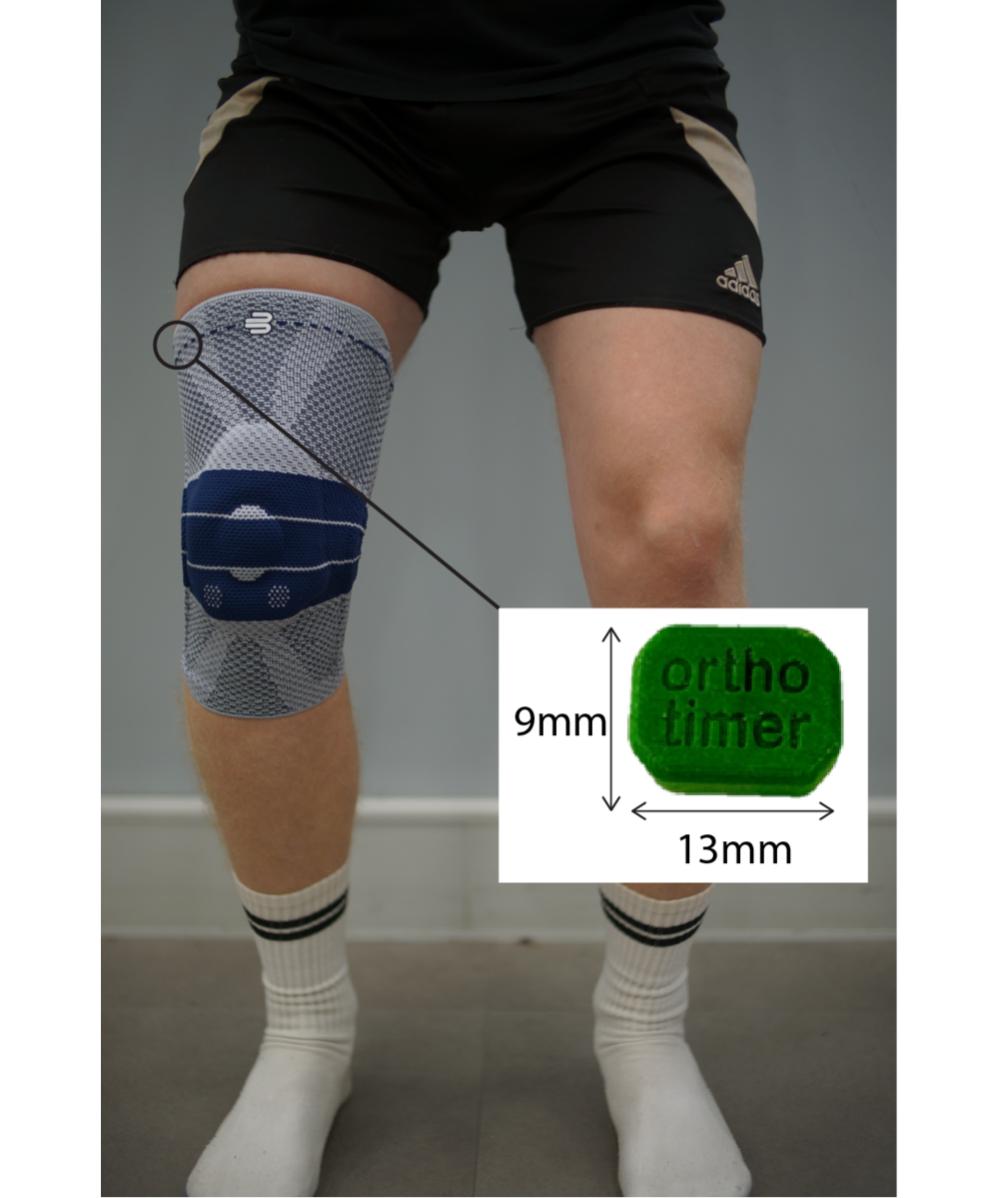
The slim-fit knee brace (GenuTrain; Bauerfeind, Germany) and temperature sensor (Orthotimer; Balingen, Germany).

This study is a secondary analysis of a 6-week feasibility trial (ACTRN12623001027606) [9] investigating a slim-fit knee brace in 21 young adults with a symptomatic knee following ACLR. No formal power calculation was performed for this exploratory analysis, consistent with our previous investigations [10], which includes only the subset of participants allocated to the brace group. Eligibility criteria included: (1) 1-8 years post-ACLR; (2) aged 18-45 years; and (3) a symptomatic knee (mean score <80/100 from the 4 Knee injury and Osteoarthritis Outcome Score subscales [KOOS_4_] [11]). Exclusion criteria were: (1) knee reinjury or lower-limb surgery in last 3 months; (2) routine use of a knee brace in the last 3 months; (3) received treatment for their index knee in the last month; (4) another condition affecting physical function; (5) pregnant; or (6) unable to understand English. For this analysis, 14 participants randomized to the brace group were included. Participants were advised to wear the brace for at least 1 hour per day, especially during knee-aggravating activities. Self-reported wear time over the intervention period was measured via a daily log on a smartphone application or paper. Several reminders were sent if a day was missed. Objective wear time was recorded via a small, commercially available temperature sensor (Orthotimer; Balingen, Germany; 9 mm×13 mm×4.5 mm) embedded in the proximal region on the side of the knee brace to ensure it was not noticeable to participants (Figure 1). The sensor uses a lithium dry cell battery (3.0 V/5.5 mAh) with a lifespan of >18 months, is waterproof, can withstand high pressures and temperatures, and has up to 400 days of storage capacity. The sensor has a temperature precision of ±0.1 °Celsius, and measured temperature once every 10 minutes. Participants were not informed of the sensor to prevent biasing self-reported wear time data. Awareness of the presence of the sensor was assessed during 6-week follow-up interviews.

### Data Analysis

#### Temperature Sensor Data Processing

**Figure 2.**
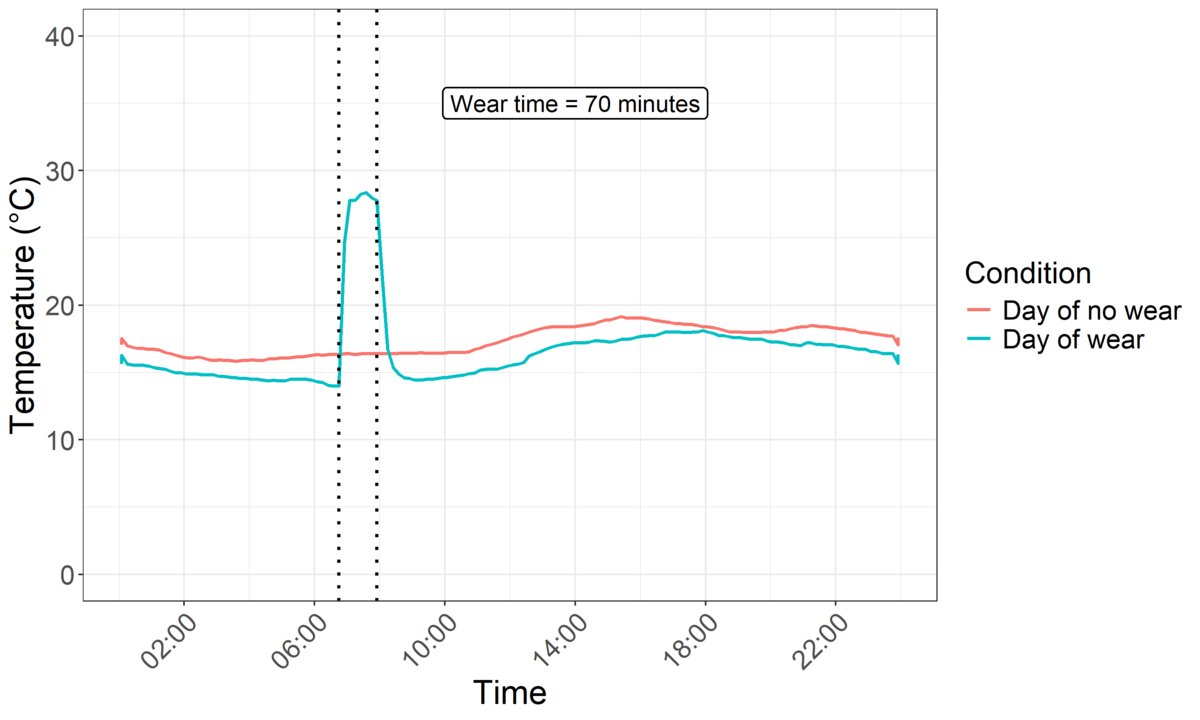
Temperature profile over 2 different days for the same participant, comparing wear (total 70 minutes) and no wear conditions.

A wear detection algorithm in Microsoft Excel (Multimedia Appendix 1) detected temperature spikes and drops to identify brace donning and doffing, respectively (Figure 2). Compared to algorithms using absolute or relative threshold values, this is the most accurate approach, and it is not influenced by ambient temperatures [8].

#### Statistical Analysis

Our analyses included (1) a description of average daily knee brace wear times (total minutes worn divided by the number of days reported in the daily log or recorded by the temperature sensor) and the number of days worn over the intervention period (days with non-zero wear time); and (2) analyses of agreement for total daily wear times, 3- and 7-day rolling averages, total wear time during the 6-week intervention period (a single cumulative value in minutes per participant, distinct from daily or rolling averages), average daily wear time over the intervention period, and the number of days worn. All data analyses were performed in R-studio (version 4.4.0). To assess agreement between the temperature sensor and self-reported wear times—accounting for repeated measures (ie, daily agreement data for each day across the intervention period for each participant)—we used concordance correlation coefficients (CCCs) and 95% limits of agreement (LoA). We considered CCC values as follows: <0.5 = poor, 0.5‐0.75=moderate, 0.75‐0.9=strong, and >0.9=excellent, consistent with common statistical practice [12]. We interpreted LoA width relative to the scale of measurement (eg, minutes per day or total days), with narrower ranges indicating less variability between methods. Sensitivity analyses assessed the impact of handling missing self-reported data, with (1) missing data imputed as “no wear” days and (2) analyses repeated excluding 1 participant who accounted for most (13 of 15) missing values.

## Results

### Participant Characteristics

**Figure 3.**
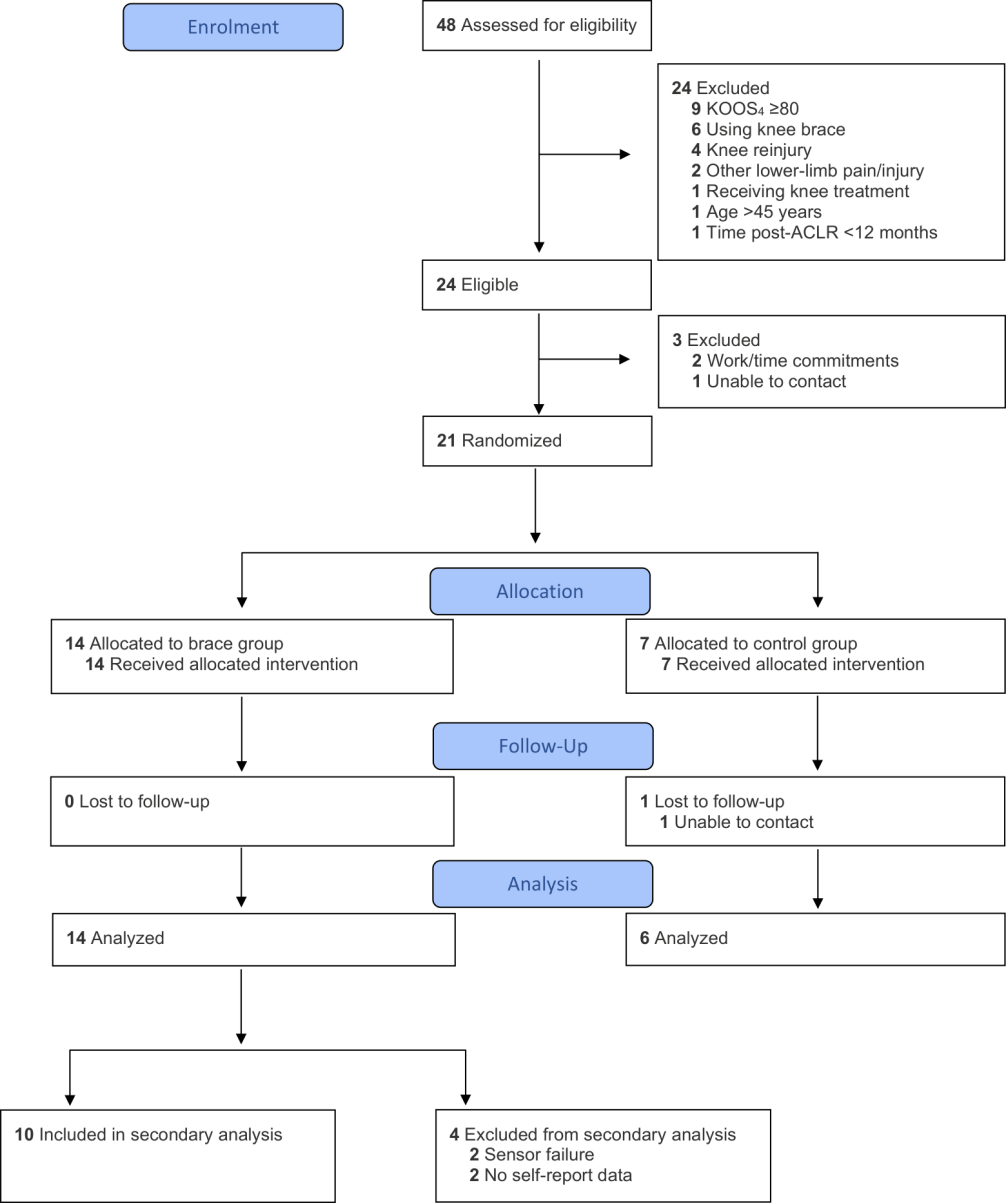
Participant flow through the secondary analysis of the 6-week feasibility trial. ACLR: anterior cruciate ligament reconstruction; KOOS_4_: 4 Knee injury and Osteoarthritis Outcome Score subscales.

Two participants were missing all temperature sensor data due to software malfunction, and 2 completed no days of self-reported data (Figure 3). The 10 remaining participants contributed to analysis (30% male [n=3]; mean age: 33, SD 6 years; body mass index: 27, SD 4 kg/m^2^; time post-ACLR: 4, SD 1 years; time between baseline and follow-up: 7, SD 2 weeks). No participant reported being aware of the brace temperature sensor during the intervention period.

### Analysis of Agreement

**Figure 4.**
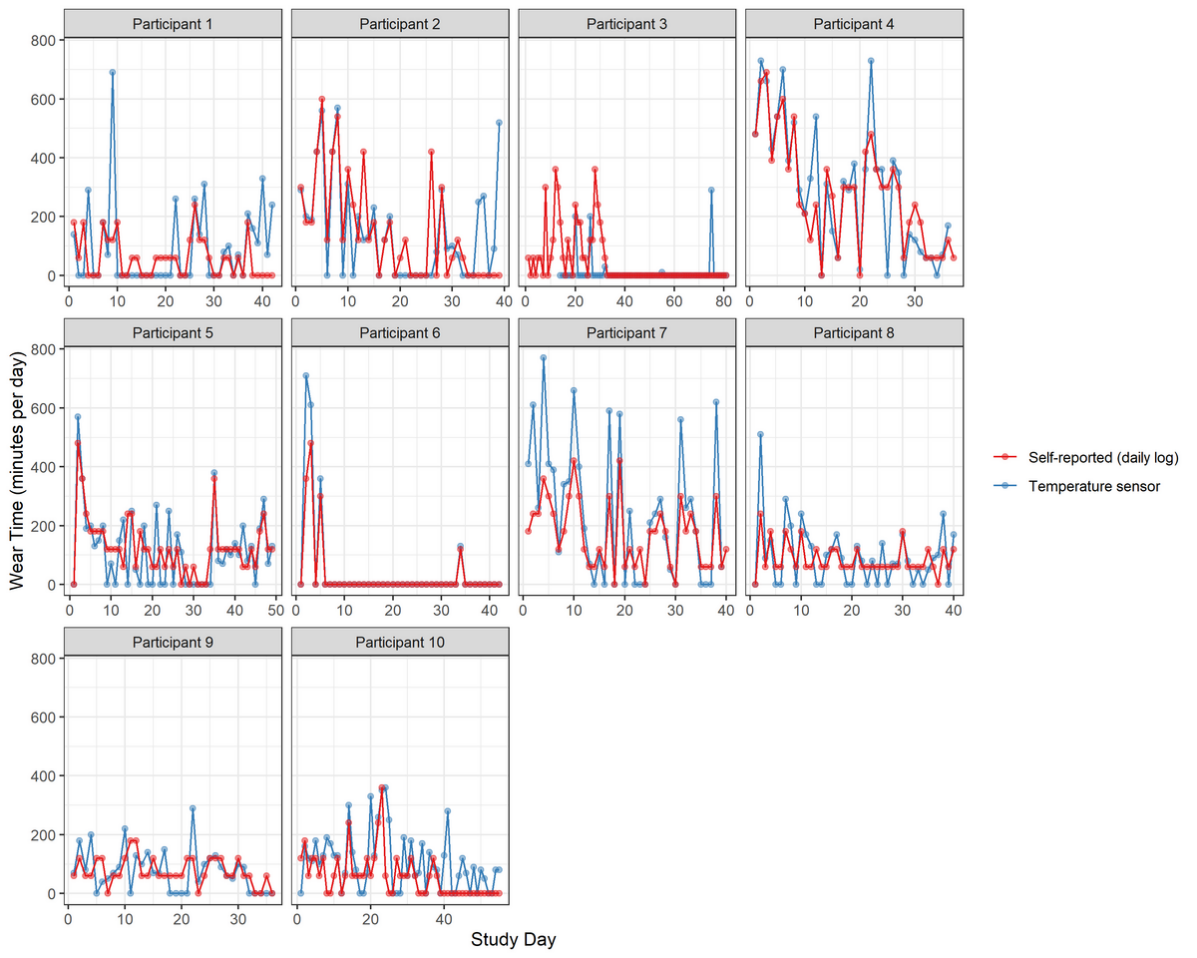
Individual participant knee brace wear time (minutes per day) comparing temperature sensor and self-report across the intervention period. The x-axis represents days of the intervention period and the y-axis represents daily wear time (minutes). Each point represents an individual participant’s wear time measured by the temperature sensor (in blue) or self-report (in red). Post-intervention testing was scheduled for 6 weeks after baseline. However, due to individual differences in participant availability, the exact timing varied slightly between participants.

**Figure 5.**
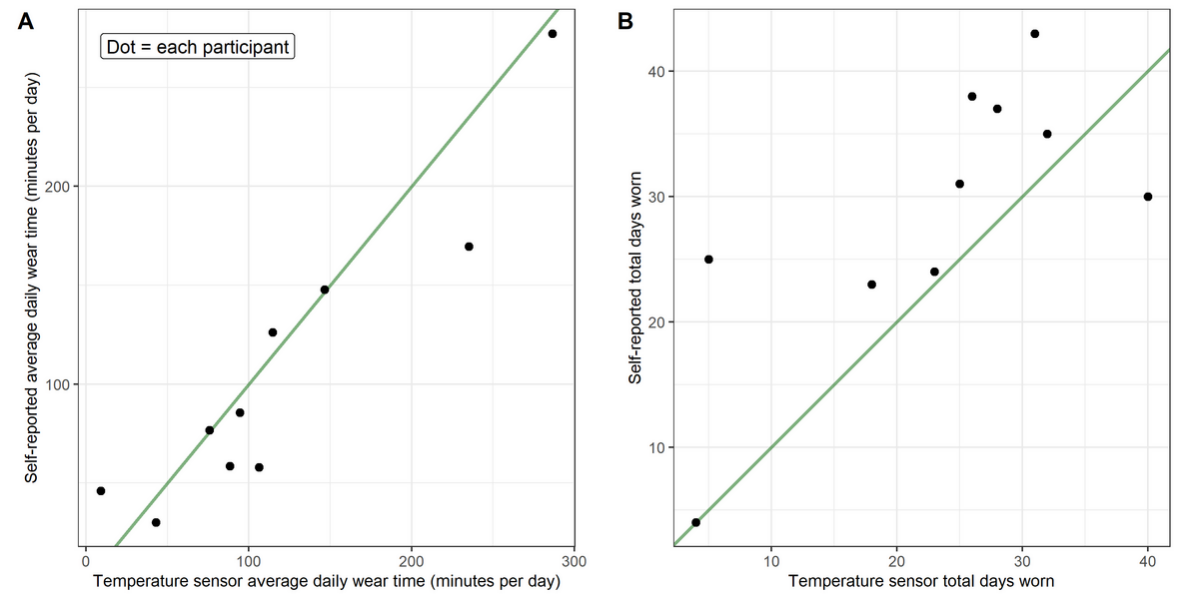
Agreement between temperature sensor and self-reported knee brace wear. Comparison of (A) average daily wear time (minutes per day, across the entire intervention period) and (B) total days worn, measured by the temperature sensor (x-axis) and self-report (y-axis). Each point represents a single participant. The green line represents the line of equality, indicating perfect agreement between methods.

There was variation between temperature sensor and self-reported data (Figure 4). Over the intervention period, 6 participants (60%) underreported the average daily knee brace wear time, and 4 (40%) overreported (mean 29, SD 24 minutes across all 10 participants) (Figure 5A). In contrast, 9 participants (90%) overreported the number of days they wore the brace, with only 1 (10%) underreporting (mean 9, SD 6 days across all 10 participants) (Figure 5B).

For repeated measures of daily wear time (ie, total minutes each day), we observed moderate agreement between the temperature sensor and self-report (CCC 0.70, 95% CI 0.58-0.79), with wide LoA (−223 to 217 minutes per day). Using 3-day rolling averages narrowed the LoA (−47 to 36 minutes per day), and 7-day rolling averages yielded the narrowest LoA (−14 to 10 minutes per day), with slightly improved CCCs (0.74, 95% CI 0.58-0.85, and 0.73, 95% CI 0.51-0.86, respectively), indicating improved agreement. For data aggregated over longer periods, agreement was strong when evaluating total wear time across the entire intervention period, with a higher CCC (0.84, 95% CI 0.50-0.95). LoA were wider however (−3065 to 1474 minutes), due to the larger scale of total minutes across the entire intervention period. When expressed as a daily average wear time, the CCC was excellent (0.92, 95% CI 0.73-0.98) and LoA narrowed (−68 to 32 minutes), though remained relatively wide, indicating substantial individual participant variability between self-reported and sensor-based measures. For the total number of days worn, the CCC was moderate (0.64, 95% CI 0.15-0.88) and LoA was wide (−10 to 22 days). Sensitivity analyses with imputed missing data or excluding 1 participant slightly altered LoA but not CCCs (Multimedia Appendix 2).

## Discussion

Our exploratory study highlights discrepancies between self-reported and temperature sensor-measured knee brace wear time. While daily knee brace wear time from the temperature sensor and daily log measures demonstrated moderate correlation, the wide LoA reflected high variability and poor accuracy in day-to-day self-reporting. Two participants (14%) failed to complete their daily logs, further highlighting the limitations of relying on self-reported knee brace wear times.

Averaging wear time over the entire intervention period, or using 3- and 7-day rolling averages, improved agreement between the temperature sensor and self-reported measures, likely due to recall bias being washed out over time. Indeed, averaging daily wear time across the entire intervention period showed excellent agreement, emphasizing that self-reports are more accurate when viewed as long-term averages, smoothing discrepancies in day-to-day reporting. This supports the common approach of reporting average wear time per day across a study period [2,13-15], which our results suggest is the optimal method for increasing accuracy of self-reported wear time estimates. However, we found poor agreement between the temperature sensor and self-reporting when knee brace wear time was assessed based on the number of days worn, with participants tending to overreport. Previous studies show that participants tend to overestimate knee brace wear time [16], with the same phenomenon observed with other interventions (eg, exercise, footwear, braces) [3,17,18], perhaps due to response or social desirability bias, where participants may overreport adherence to appear more compliant. Similar discrepancies between self-reported and objectively measured adherence have been reported in broader health interventions, including physical activity monitoring [19] and medication adherence programs, such as in antiretroviral therapy [20]. These findings further emphasize the importance of objective adherence measurement (eg, temperature sensors) to accurately measure knee brace wear time.

While CCCs indicate moderate-excellent agreement, the wide LoA reflect substantial variability. Our results indicate that daily self-reported wear time could be expected to differ from sensor-measured wear time by up to 3.5 hours higher or lower per day (LoA −223 to 217 minutes), potentially misjudging adherence and influencing clinical or research decisions if they are based on single day wear times. However, aggregating wear over 7-day periods reduced this discrepancy (LoA −14 to 10 minutes), with self-reported wear time underestimating sensor-measured wear by up to 14 minutes or overestimating it by up to 10 minutes. Using the average daily wear across the entire intervention period reduced variability (LoA −68 to 32 minutes), with self-reported wear time underestimating sensor-measured wear by up to 68 minutes or overestimating it by up to 32 minutes, suggesting that longer-term averages smooth day-to-day reporting errors.

In clinical settings, where adherence to knee brace wear is important for treatment outcomes [3], reliance on self-reported wear time may misclassify adherence and lead to suboptimal management. From a research perspective, inaccurate adherence measurement may bias assessments of knee brace intervention effectiveness [3]. However, these findings are exploratory and should be interpreted with caution given the small sample size. Future studies should validate temperature-based wear detection for knee braces and evaluate adherence methods in larger samples.

Key limitations should be acknowledged. Firstly, the temperature sensor, used in this study, has not been specifically validated for knee braces, though it has demonstrated high accuracy in detecting wear time in footwear and orthotics compared to reference standards, such as cameras or smartphone applications [7,8]. Knee-specific factors (eg, skin contact, brace positioning, local sweating) could influence temperature measurements and thus sensor accuracy. While we expect sensor performance to be broadly transferable, direct validation in knee braces would strengthen confidence in these findings. Secondly, while temperature spikes were used to infer brace donning and doffing, environmental factors may have influenced temperature changes (eg, transitioning to a hot car or air-conditioned room). Finally, the small sample size (n=10) limits the generalizability of our findings and likely contributes to the wide limits of agreement observed. As such, our results should be interpreted as preliminary and hypothesis-generating rather than definitive estimates of adherence accuracy.

These exploratory findings highlight potential limitations of relying solely on self-reported knee brace wear. Future studies could consider objective measures of knee brace wear adherence (eg, temperature sensors) to complement self-reported data and mitigate the impact of recall and response bias. In settings where objective monitoring is not available, averaging self-reported wear time across the entire intervention period could improve accuracy compared to day-to-day reporting.

## Supplementary material

10.2196/79725 Multimedia Appendix 1 Wear detection algorithm.

10.2196/79725 Multimedia Appendix 2 Sensitivity analyses.

10.2196/79725 Checklist 1 CONSORT-eHEALTH checklist (V 1.6.1).

## Abbreviations

ACLR: anterior cruciate ligament reconstruction
CCC: concordance correlation coefficient
KOOS_4_: 4 Knee injury and Osteoarthritis Outcome Score subscales
LoA: limits of agreement
