# Supplementary material for: Accuracy in the Estimation of Self-Reported Knee Brace Wear Time in Young Adults With a Symptomatic Knee Following ACL Reconstruction: Secondary Analysis of a Pilot Randomized Controlled Trial
Source: JMIR Rehabil Assist Technol. 2026 May 27;13:e79725. doi: 10.2196/79725 (PMC13215634; doi:10.2196/79725)
Supplement: Multimedia Appendix 1 [file rehab-v13-e79725-s001.docx]

**Multimedia Appendix 1.** Wear detection algorithm

The wear detection algorithm, used on Microsoft Excel (Microsoft Corporation, Redmond, WA, USA, Excel 365, Version 2505), analysed temperature data recorded every 10 minutes. Temperature changes were calculated by subtracting each reading from the previous one. Spikes indicated brace donning, while drops indicated doffing. Only spikes or drops distinguishable from smaller background temperature variations were used, as opposed to applying fixed cutoff values. Wear data for each participant was manually checked and extracted independently by two researchers (MS, HS), and disagreements discussed until consensus, or with a third researcher available to resolve conflicts (AC, BM). In cases of disagreement, temperature values from the sensor were cross-checked with minute-by-minute, dry bulb, ambient temperature data from a local weather station close to La Trobe University, obtained from the Australian Bureau of Meteorology.


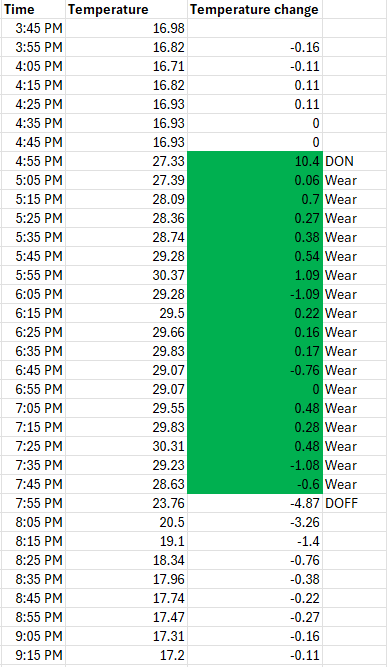


**Supplementary Figure 1:** Example of the wear detection algorithm applied in Microsoft Excel, showing 180 minutes of brace wear time.
