# Supplementary material for: Accuracy in the Estimation of Self-Reported Knee Brace Wear Time in Young Adults With a Symptomatic Knee Following ACL Reconstruction: Secondary Analysis of a Pilot Randomized Controlled Trial
Source: JMIR Rehabil Assist Technol. 2026 May 27;13:e79725. doi: 10.2196/79725 (PMC13215634; doi:10.2196/79725)
Supplement: Multimedia Appendix 2 [file rehab-v13-e79725-s002.docx]

# **Multimedia Appendix 2.** Sensitivity analyses

i) Sensitivity analyses for agreement between self-reported and sensor-measured wear time (CCC)

| Measure | Raw (95% CI) | Imputed daily log (95% CI) (not imputed sensor) | Excluded participant 1011^1^ (95% CI) (no imputation) | Imputed both and excluded participant 1011 (95% CI) | Imputed both and include participant 1011 (95% CI) |
| --- | --- | --- | --- | --- | --- |
| Daily wear time (minutes) | 0.70 (0.59 to 0.79) | 0.71 (0.59 to 0.81) | 0.73 (0.62 to 0.82) | 0.73 (0.62 to 0.81) | 0.71 (0.56 to 0.80) |
| 3-day rolling averages | N/A^2^ | N/A^2^ | N/A^2^ | 0.84 (0.72 to 0.91) | 0.74 (0.58 to 0.85) |
| 7-day rolling averages | N/A^2^ | N/A^2^ | N/A^2^ | 0.89 (0.76 to 0.95) | 0.73 (0.51 to 0.86) |
| Total wear time | N/A^2^ | N/A^2^ | N/A^2^ | 0.88 (0.59 to 0.97) | 0.84 (0.50 to 0.95) |
| Average daily wear time | N/A^2^ | N/A^2^ | N/A^2^ | 0.92 (0.71 to 0.98) | 0.92 (0.73 to 0.98) |
| Total days worn | N/A^2^ | N/A^2^ | N/A^2^ | 0.74 (0.26 to 0.92) | 0.64 (0.15 to 0.88) |

^1^ = Participant was excluded who accounted for the most missing values (13 of 15); ^2^ = All rolling calculations require imputing first

ii) Sensitivity analyses for agreement between self-reported and sensor-measured wear time (95 % LoA and mean bias)

| Measure | Raw | Imputed daily log (not imputed sensor) | Excluded participant 1011^1^ (no imputation) | Imputed both and excluded participant 1011 | Imputed both and include participant 1011 |
| --- | --- | --- | --- | --- | --- |
| Daily wear time (minutes) | -223 to 217  Bias = -3 (-39 to 34) | -216 to 187  Bias = -14 (-35, 6) | -223 to 198  Bias = -13 (-42, 17) | -225 to 188  Bias = -18 (-38, 1) | -218 to 194  Bias = -12 (-34, 10) |
| 3-day rolling averages | N/A^2^ | N/A^2^ | N/A^2^ | -47 to 36  Bias = -5 (-12, 1) | -48 to 41  Bias = -4 (-11, 4) |
| 7-day rolling averages | N/A^2^ | N/A^2^ | N/A^2^ | -14 to 10  Bias = -2 (-5, 1) | -15 to 13  Bias = -1 (-4, 2) |
| Total wear time | N/A^2^ | N/A^2^ | N/A^2^ | -3065 to 1474  Bias = -796 (-1685, 94) | -3592 to 2758  Bias = -417 (-1576, 742) |
| Average daily wear time | N/A^2^ | N/A^2^ | N/A^2^ | -68 to 32  Bias = -18 (-37, 1) | -70 to 45  Bias = -13 (-34, 9) |
| Total days worn | N/A^2^ | N/A^2^ | N/A^2^ | -9 to 18  Bias = 4 (-1, 10) | -10 to 22  Bias = 6 (0, 12) |

^1^ = Participant was excluded who accounted for the most missing values (13 of 15); ^2^ = All rolling calculations require imputing first
